# Atraumatic Compartment Syndrome Presenting As Wrist Drop: A Case Report

**DOI:** 10.7759/cureus.97514

**Published:** 2025-11-22

**Authors:** Grace-Anna Perry, Rachel E Aliotta, Briana Miller, Zachary Pacheco, Erin F Shufflebarger

**Affiliations:** 1 Department of Emergency Medicine, University of Alabama at Birmingham, Birmingham, USA; 2 Department of Orthopedic Surgery, University of Alabama at Birmingham, Birmingham, USA; 3 Department of Emergency Medicine, Oregon Health and Science University School of Medicine, Portland, USA

**Keywords:** clinical case, compartment syndrome, emergency medicine, radial nerve palsy, rhabdomyolysis, wrist drop

## Abstract

Wrist drop, a common motor deficit seen with radial neuropathy, is typically a result of nerve damage from acute trauma or external compression. Diagnosis is initially suspected by clinical examination, and initial management consists of splinting the wrist, physical therapy, and referral for consideration of outpatient surgical management if it doesn't resolve with conservative measures. While this is commonly managed in the outpatient setting, the emergency physician should keep a broad differential when evaluating patients with wrist drop, as there are rare causes of wrist drop that may require emergent surgical management. In this report, we discuss a case of wrist drop due to compartment syndrome.

We present a case of a male in his early 30s who presented to the emergency department (ED) with left upper extremity numbness and weakness noticed upon awakening. Examination revealed tense compartments in the left forearm with associated wrist drop. Laboratory workup revealed rhabdomyolysis with multi-organ failure, and the patient was taken for emergent fasciotomy with orthopedic surgery.

This report highlights a case of acute compartment syndrome in a critically ill patient who initially presented to the ED with wrist drop. The case demonstrates the need for emergency physicians and other acute care clinicians to keep a broad differential in patients presenting with acute peripheral neuropathies. This rare cause of radial neuropathy warrants consideration, as prompt recognition, diagnosis, and emergent management of compartment syndrome are needed to prevent life and limb-threatening morbidity and mortality.

## Introduction

Wrist drop, or inability to extend at the wrist, is the primary clinical feature associated with radial neuropathy [1]. Radial neuropathies are commonly due to damage or compression of the radial nerve. Traumatic radial nerve injuries can be seen with penetrating trauma and humerus fractures. Radial neuropathies due to external compression of the nerve are also common. This includes compression at the axilla or "crutch palsy", compression in the upper arm or "Saturday night palsy", typically associated with intoxication, or superficial compression at the wrist or "cheiralgia paresthetica" from tight handcuffs or wristwatches [1-2]. In the acute care setting, this diagnosis is typically suspected after clinical examination, and initial management generally consists of splinting the wrist and physical therapy with referral for surgical consideration if this doesn't resolve with time and conservative measures [2].

While most causes of radial neuropathy are relatively benign and resolve with conservative management, the emergency physician should maintain a broad differential when evaluating patients with wrist drop, as there are rare causes of wrist drop that require emergent intervention. One such cause is compartment syndrome, a surgical emergency characterized by increased tissue pressure within an enclosed fascial compartment, which leads to ischemic injury of muscles and nerves [3,4]. When compartment syndrome involves the forearm, compression of the radial nerve can occur, resulting in wrist drop. The majority of compartment syndrome cases arise secondary to traumatic injury [3]. Prompt recognition and fasciotomy are critical, as delay in diagnosis or management can lead to devastating complications including irreversible motor deficits, contractures, amputation, rhabdomyolysis, renal failure, or even death [3,4].

In this report, we discuss the rare case of radial nerve palsy due to atraumatic compartment syndrome in a critically ill patient, highlighting the importance of considering compartment syndrome in the differential diagnosis of acute peripheral neuropathy.

## Case presentation

A male in his early 30s with a history of non-intravenous polysubstance use presented to the emergency department (ED) with primary concern for left upper extremity weakness and numbness noted upon awakening. He also reported some tearing of the left eye, and a friend at bedside was concerned for a new facial asymmetry. 

**Figure 1:**
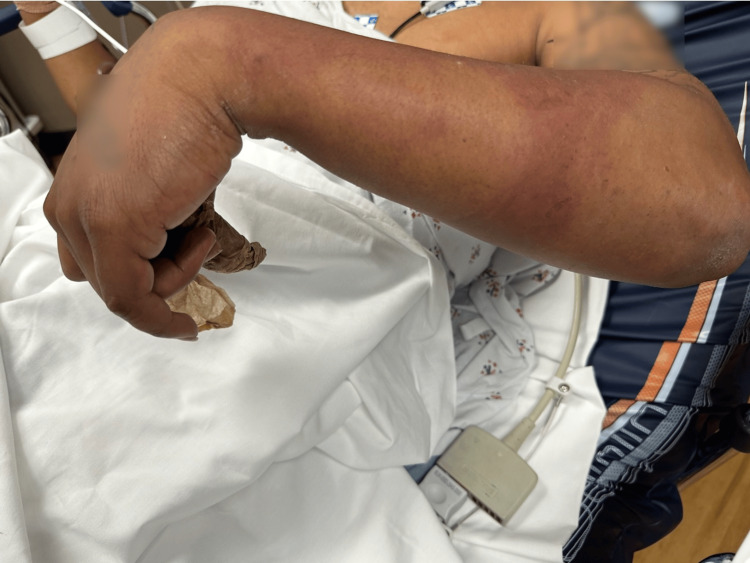
Left upper extremity examination revealing streaky discoloration of the forearm and inability to extend at his wrist. Written consent was obtained from the patient for the acquisition and use of this image, which has been de-identified.

On arrival to the ED, he appeared generally ill and uncomfortable. He was afebrile and hemodynamically stable with a heart rate of 97 beats per minute, blood pressure of 102/68 mm/Hg, and temperature of 97.4 degrees Fahrenheit. His respiratory rate was 18 respirations per minute, and his oxygen saturation was 96% on room air. He had tearing of the left eye without conjunctival erythema or corneal abrasion on fluorescein staining, and mild eyelid swelling was noted, concerning for possible ptosis. His extremity examination revealed streaky discoloration of the left forearm and inability to extend at the wrist on visual examination (Figure 1). His forearm was notably swollen with tense compartments. His left upper extremity neurovascular examination was notable for a warm and well-perfused extremity with a faint palpable radial pulse, weakness at the wrist, but preserved range of motion at the elbow and shoulder. A detailed sensory examination was limited, though he had some reported decreased sensation in the left hand. 

A broad initial differential diagnosis was considered, including, but not limited to, acute compartment syndrome, acute stroke, aortic dissection, infectious process including necrotizing fasciitis, fracture, acute extremity venous/arterial occlusion, and isolated peripheral neuropathy. Given that the initial evaluation revealed an acute neurological deficit involving the extremity accompanied by mild facial asymmetry, a "code stroke" was activated in accordance with institutional guidelines to evaluate for possible large vessel occlusion, as the patient was within the window for potential thrombectomy. However, the patient's CT imaging, neurology evaluation, and subsequently inpatient magnetic resonance imaging were reassuring against acute stroke. Orthopedic surgery was also consulted immediately, given the clinical concern for acute compartment syndrome, an alternate explanation for the patient's acute deficit. Given the high suspicion for compartment syndrome based on the physical exam, intracompartmental pressure assessment was deferred, and surgical consultation was initiated before laboratory or imaging results were available. 

Initial laboratory results (Table 1) revealed elevated creatinine kinase and elevated creatinine, concerning for rhabdomyolysis and acute renal failure with associated non-hemolyzed hyperkalemia. Intravenous fluid resuscitation was initiated, and the patient also received medications for hyperkalemia, including calcium gluconate and insulin with dextrose-containing fluids. Significant leukocytosis and elevated lactic acid were also noted, prompting broad-spectrum antibiotic administration. Laboratory testing also revealed elevated liver enzymes and troponin, suggesting acute hepatic failure and myocardial injury. Computed tomography (CT) of the forearm revealed non-specific findings, including heterogeneous muscle enhancement throughout the forearm and pooling edema between the anterior/lateral compartments without soft tissue gas appreciated. 

**Table 1:** Pertinent laboratory values

Laboratory test	Patient value	Reference range	Unit
Sodium	137	133-145	mMol/L
Potassium	6.8	3.1-5.1	mMol/L
Chloride	99	97-108	mMol/L
Glucose	114	70-100	mg/dL
Blood Urea Nitrogen (BUN)	35	5-22	mg/dL
Creatinine	4.3	0.7-1.3	mg/dL
Lactic acid	3.3	0.5-2.2	mMol/L
White blood cell (WBC)	26.8	4-11	x10^3 ^cm^3^
Creatinine kinase	84,966	35-250	U/L
Aspartate aminotransferase (AST)	1,151	12-39	U/L
Alanine transaminase (ALT)	463	7-52	U/L
Troponin, high-sensitivity	1,053	3-20	ng/L
Erythrocyte sedimentation rate (ESR)	31	0-10	mm/hr
C-reactive protein (CRP)	42.89	0-10.9	mg/L

After prompt evaluation by the surgery team, the patient was taken for emergent fasciotomy/decompression of the left forearm. Intraoperatively, the muscles of the upper arm posterior compartment and forearm dorsal compartments initially appeared dusky, but the color improved following fascial release. There was no evidence of necrotizing infection. He was discharged after a 15-day hospital stay. After three months of hand therapy and close follow-up, he remarkably regained full motor function of his upper extremity.

## Discussion

This patient presented with atraumatic left upper extremity sensory and motor deficits, including wrist drop, and was found to have compartment syndrome requiring emergent fasciotomy. Typically, radial neuropathies are attributed to nerve injury from trauma or compression and can be managed on an outpatient basis, with non-emergent surgical intervention only considered after trial of conservative measures [2]. Wrist drop from more emergent causes like this, atraumatic compartment syndrome is rare; however, the emergency physicians should maintain clinical suspicion as prompt recognition and emergent surgical management are critical to ensuring the best chance of clinical recovery.

Compartment syndrome is a critical condition that occurs due to increased tissue pressure within an enclosed fascial area. Increased compartment pressure leads to reduced perfusion/oxygenation and compromised function of nerves and muscles in that space [3]. This is most commonly a result of trauma, with over 80 percent of acute compartment syndrome associated with acute extremity fractures [5]. Acute compartment syndrome can also result from other means, including tight casts/dressings, burns, intravenous extravasation, bleeding disorders, intensive use of muscles during exercise, or seizure [3]. Prolonged immobilization with sustained pressure on a single limb represents another rare but recognized cause of atraumatic compartment syndrome that may be pertinent to our case [4]. This mechanism, sometimes referred to as "found down" compartment syndrome, has been reported with increasing frequency in recent years and is often associated with substance use [6].

Compartment syndrome is suspected by clinical examination, with the classic presentation of the Five P's: pain, pulselessness, paresthesia, paralysis, and pallor, though these are notably late findings of this diagnosis [7]. Pain is typically described as severe and "out of proportion to the insult or injury" [4]. If early compartment syndrome is suspected, serial examinations are recommended due to the propensity for rapid progression [7]. If the diagnosis is uncertain, intracompartmental pressure measurement can be acquired to aid in diagnosis [7], though these aren't required as compartment syndrome is a clinical diagnosis [4]. Radiographs should be obtained if a fracture is suspected, and creatine kinase (CK) levels may suggest muscle breakdown or rhabdomyolysis [7]. One study found that an elevated CK level was correlated with a higher risk of poor outcomes, with CK above 300 U/L proposed as a sensitive predictor and CK above 10,000 U/L proposed as a specific predictor of poor outcomes in patients with acute compartment syndrome of the forearm [8]. Although CT imaging is not routinely used in the diagnosis of acute compartment syndrome, non-specific findings of muscle enhancement and edema, similar to those in our case, have been reported previously [9].

Compartment syndrome is a surgical emergency that leads to ischemia and tissue necrosis if untreated, and prompt diagnosis and immediate surgical consultation for fasciotomy are critical [7]. Long-term outcomes depend primarily on the timing of diagnosis and treatment. Prompt surgical management generally leads to better patient outcomes. When fasciotomy is completed within six hours of onset, full recovery of limb function is generally expected [7]. Residual nerve damage can be seen after six hours, and when fasciotomy is done within 12 hours, approximately two-thirds of patients recover limb function [7]. In cases with significantly delayed operative management, the limb may require an amputation [7]. Post-operative management typically includes physical and occupational therapy, wound care, and pain control, with management best done with a multidisciplinary approach and close follow-up.

## Conclusions

This report highlights a case of acute compartment syndrome in a critically ill patient who initially presented to the ED with wrist drop. The case demonstrates the need for emergency physicians and other acute care clinicians to keep a broad differential in patients presenting with acute peripheral neuropathies. This rare cause of radial neuropathy warrants consideration, as prompt recognition, diagnosis, and emergent management of compartment syndrome are needed to prevent life- or limb-threatening morbidity/mortality.
